# Use of religious and spiritual complementary practices among Chinese patients with schizophrenia: prevalence, perceived helpfulness, and associated factors

**DOI:** 10.3389/fpsyt.2026.1948994

**Published:** 2026-10-21

**Authors:** Mao Tang, Hong Liu, Bao-Liang Zhong

**Affiliations:** 1 Department of Psychiatry, Affiliated Wuhan Mental Health Center, Tongji Medical College of Huazhong University of Science and Technology, Wuhan, China; 2 Department of Psychiatry, Wuhan Mental Health Center, Wuhan, China

**Keywords:** China, complementary therapies, perceived helpfulness, religion, schizophrenia, spirituality

## Abstract

**Background:**

Religious and spiritual practices are increasingly recognised as complementary approaches for managing severe mental illnesses, but their use among patients with schizophrenia in China remains poorly understood.

**Objectives:**

To investigate religious affiliation, religious, spiritual, or folk healing practices, perceived helpfulness, and associated factors among Chinese patients with schizophrenia.

**Methods:**

A total of 299 clinically stable outpatients with schizophrenia were consecutively recruited from a tertiary psychiatric hospital in Wuhan, China. A standardised questionnaire assessed religious affiliation, use of religious, spiritual, or folk healing practices for managing mental illness, and perceived helpfulness. Classification tree analyses were conducted to identify factors associated with practice use and perceived helpfulness.

**Results:**

Overall, 36.5% of participants reported a religious affiliation, with Buddhism being the most common. More than half of participants (57.2%) had used religious, spiritual, or folk healing practices to manage their illness. Among users, 69.6% perceived these practices as helpful. The most commonly reported practices were Zen meditation (44.8%) and scripture chanting and religious rituals (24.1%). Classification tree analyses identified several factors associated with practice use, including belief in reincarnation and karma, non-conventional health beliefs, educational level, age, occupation, and family members’ supernatural beliefs. Religious affiliation and family members’ supernatural beliefs were additionally associated with perceived helpfulness.

**Conclusion:**

Religious and spiritual complementary practices were common among Chinese outpatients with schizophrenia and were often perceived as helpful. These findings highlight the relevance of considering patients’ religious and spiritual practices in culturally sensitive psychiatric assessment and care.

## 1 Introduction

Religion and spirituality exert a significant, albeit complex, influence on the lives of many individuals with severe mental illnesses, including schizophrenia (1). For many patients and families, religious or spiritual beliefs may serve as important personal and social resources, offering solace, meaning, hope, and a culturally familiar framework for coping with the overwhelming experiences associated with psychotic disorders (2). For others, however, religious beliefs and practices may become intertwined with psychopathology, exacerbate distress, or shape interpretations of hallucinations, delusions, and other psychotic symptoms in ways that complicate clinical assessment and care (3). This dual potential has attracted growing interest in psychiatric research and highlights religion and spirituality as clinically relevant dimensions of patients’ illness experiences and coping (4).

In Western countries, a substantial body of literature has examined the relationship between religion, spirituality, and schizophrenia. Studies have consistently shown that many patients use religion or spirituality to cope with their illness, with some estimates suggesting proportions as high as 80% (5). Positive religious coping, including prayer, participation in religious communities, and meaning-making through faith, has been associated with enhanced hope and purpose, reduced distress, greater social support, and improved quality of life (3, 6, 7). Religious communities may provide emotional and practical support, while faith can offer a “meaning-endowing framework” through which illness experiences are interpreted and integrated into personal life narratives (8). At the same time, the role of religion and spirituality is not uniformly positive. Negative religious coping may contribute to spiritual distress, fear, guilt, or self-blame and has been associated with greater symptom burden or poorer psychosocial outcomes (9, 10), while the phenomenological overlap between psychotic symptoms and religious or spiritual experiences may complicate the distinction between culturally accepted experiences and symptoms requiring clinical intervention (11). Although this literature provides an important foundation, its findings may not be directly applicable to non-Western contexts, particularly China. Whereas Western research has often been conducted in contexts shaped by relatively institutionalized and exclusive monotheistic traditions, Chinese spiritual life is frequently characterised by a syncretic, pragmatic, and non-exclusive combination of Buddhism, Daoism, Confucian traditions, ancestor veneration, and folk religious practices (12, 13). Formal religious affiliation may be relatively low, yet beliefs and practices concerning karma and reincarnation, spirit influence, yin-yang and qi, ritual healing, and other culturally embedded understandings remain part of everyday religious and cultural life for many people (14, 15). Consequently, schizophrenia and related psychotic experiences may be interpreted not only through biomedical frameworks but also through supernatural, moral, relational, or cosmological understandings (16–18).

In the Chinese context, these culturally embedded beliefs may be expressed not only in explanations of mental illness but also in concrete religious and spiritual practices used by patients and their families in responding to illness (19). Some individuals may draw on prayer, meditation, ritual activities, temple-based practices, or other culturally familiar practices as part of their efforts to cope with and manage mental illness (20, 21). Accordingly, understanding religious and spiritual involvement requires attention not only to formal religious affiliation but also to whether such practices are actually used in illness management.

Beyond practice use itself, patients’ perceived helpfulness of these practices provides an additional perspective on how they experience and value them. In schizophrenia research and recovery-oriented psychiatry, patient-reported perspectives complement clinician-rated outcomes by capturing subjective dimensions of recovery, including perceived benefit, meaning, and quality of life (22–24). In the present study, perceived helpfulness was therefore conceptualized as a patient-reported appraisal of subjective benefit rather than as evidence of symptom reduction or clinical efficacy. Such appraisals may provide insight into the personal and cultural significance that patients attach to religious and spiritual practices and into dimensions of their illness experience that may receive limited attention in routine psychiatric care.

Previous studies in Chinese cultural contexts have examined religiosity, religious or culturally shaped psychotic experiences, traditional and magico-religious healing practices, and the role of spirituality in treatment and recovery among people with schizophrenia (14, 25–28). However, this evidence remains relatively limited and geographically concentrated in specific settings and clinical populations, with prior research focusing mainly on religious beliefs, symptom-related experiences, and broader treatment, coping, or recovery issues (5, 26, 27). A systematic review of religion, spirituality, and supernatural-belief research in mainland China further showed that most studies used cross-sectional or case-control designs, and many relied primarily on religious affiliation as a measure, leaving more detailed dimensions of religious and spiritual involvement less well characterised (29). Less is known about the use of specific religious and spiritual practices for managing mental illness, patients’ perceived helpfulness of these practices, and the factors associated with practice use and perceived helpfulness (29, 30).

Therefore, this study aimed to address this gap by exploring the use of religious and spiritual practices for managing mental illness among patients with schizophrenia in China. Specifically, we sought to describe religious affiliation and the prevalence and patterns of practice use, assess patient-reported perceived helpfulness among those who had used these practices, and explore factors associated with both practice use and perceived helpfulness using classification tree analyses. By characterizing how religious and spiritual practices are used and perceived by patients with schizophrenia, as well as the factors associated with these experiences, this study aimed to provide an empirical basis for more culturally responsive and spiritually informed psychiatric care in China.

## 2 Methodology

### 2.1 Setting and participants

This cross-sectional survey was conducted between March and May 2024. Participants were consecutively recruited from the outpatient department of Wuhan Mental Health Center, a large psychiatric specialty hospital in south-central China. Consecutive sampling was used to reduce selection bias and to reflect the clinical population of patients with schizophrenia attending routine outpatient services during the study period.

Eligible participants were patients who met the following inclusion criteria: 1) aged 18–60 years, the upper age limit of 60 years was prespecified to restrict the study population to non-geriatric adults and reduce heterogeneity related to age-associated cognitive and medical comorbidities; 2) had a confirmed diagnosis of schizophrenia according to the International Classification of Diseases, 10th Revision, ICD-10; and 3) were clinically stable and able to complete a questionnaire-based interview. Clinical stability was determined by the treating psychiatrist based on the absence of an acute psychotic episode requiring emergency intervention or inpatient admission at the time of recruitment. Patients were excluded if they had severe physical illness, marked cognitive or communication difficulties, or acute psychotic symptoms that precluded participation in the questionnaire interview. Accordingly, the study population represents clinically stable outpatients and does not encompass the full clinical spectrum of patients with schizophrenia.

Wuhan Mental Health Center is the largest psychiatric specialty hospital in south-central China and provides mental health services to approximately 14 million residents in Wuhan and surrounding cities. In 2024, the center recorded approximately 440,000 outpatient visits. The hospital therefore represents an important clinical setting for examining religious, spiritual, or folk healing practices among patients with schizophrenia in central China.

The study was conducted in accordance with the ethical principles of the Declaration of Helsinki and its subsequent amendments, as well as relevant ethical regulations in China. The study protocol was approved by the Medical Ethics Committee of the Wuhan Mental Health Center, approval number KY2024.0227.01.

All participants were informed about the purpose of the study, the voluntary nature of participation, confidentiality, and their right to withdraw at any time without affecting their medical care. Before enrollment, the treating psychiatrist confirmed that the patient was clinically stable and assessed the patient’s capacity to provide informed consent. Written informed consent was obtained from all participants before the questionnaire interview. Guardian consent was obtained for 12 participants (4.0%) when the treating psychiatrist judged that psychiatric symptoms raised concerns about fully independent informed consent, while the participant remained capable of understanding and responding to the questionnaire. Guardian consent served as an additional ethical safeguard and did not involve proxy completion of the questionnaire. Data were collected anonymously and handled confidentially.

### 2.2 Questionnaire and survey procedures

A standardised questionnaire was used to collect information on patients’ sociodemographic characteristics, clinical characteristics, religious or spiritual beliefs, religious, spiritual and folk healing practices, and non-conventional health beliefs. Clinical variables included age at onset, duration of schizophrenia, and family history of mental illness.

Religious affiliation was assessed using the question, “Do you have any religious beliefs?” Participants who answered “yes” were then asked, “Which religion do you primarily follow?” (13). Because formal religious affiliation may not fully capture culturally embedded spiritual or folk beliefs in China, additional questions were used to assess beliefs and practices related to karma, reincarnation, supernatural explanations, and religious, spiritual or folk healing.

To assess the use of religious, spiritual, or folk healing practices, a four-item checklist was developed based on a review of relevant literature in the Chinese context and preliminary qualitative interviews with 10 patients with schizophrenia. The initial items were reviewed by scholars with expertise in Chinese religion and culture for content relevance, cultural appropriateness, and clarity, and the wording was revised accordingly. The final checklist included four practices: 1) Zen meditation; 2) scripture chanting and religious rituals; 3) religious prayer; and 4) shamanic dance or ritual practices for exorcism. Participants were asked whether they had ever used any of these practices to treat or alleviate their mental illness. For each endorsed practice, they were further asked, “Do you think this practice is helpful for your mental illness?” with response options of “yes” or “no.”

This subjective evaluation was treated as a patient-reported perceived helpfulness outcome. It was intended to capture patients’ own views about whether these practices were helpful for their illness experience, rather than to establish clinical efficacy. Patient-reported outcomes are relevant to patient-centered and culturally sensitive care because they reflect patients’ subjective experiences, perceived benefits, and treatment-related preferences in real-world contexts (22, 31, 32).

To assess non-conventional health beliefs relevant to the Chinese cultural context, we developed a study-specific exploratory four-item scale based on prior literature on CAM-related health beliefs (33), with wording adapted to culturally familiar Chinese views of health and illness. The four items assessed beliefs that: 1) health is maintained by a vital force; 2) health reflects a balance of opposing forces; 3) the body has a natural self-healing ability; and 4) symptoms reflect a whole-body imbalance. Participants rated each item on a 7-point Likert scale ranging from 1, “absolutely agree,” to 7, “absolutely disagree.” A total score was calculated by summing the four item scores, with lower scores indicating stronger non-conventional health beliefs. The internal consistency of this study-specific four-item measure was acceptable in the current sample, with Cronbach’s α = 0.75. No formal factor analysis or validity testing was performed; therefore, the scale was treated as an exploratory measure in the present study.

Considering the culturally relevant belief in China that schizophrenia or other serious mental illness may be interpreted by some individuals as related to past-life wrongdoing, karmic retribution, or supernatural causation (34), two additional belief-related variables were assessed. First, participants were asked whether they believed in reincarnation, defined as the cycle of life and death, and karma, defined as the law of cause and effect. Second, they were asked whether any family members held supernatural beliefs about illness. Both questions used a dichotomous yes/no response format.

The questionnaire was administered by trained psychiatric nurses either before or after the patients’ routine appointments with their treating psychiatrists. Nurses received standardised instructions regarding the purpose of the study, the meaning of each questionnaire item, and procedures for obtaining informed consent and maintaining confidentiality. When patients had difficulty recalling clinical history or family-related information, accompanying family members were permitted to provide supplementary factual information. However, all questions concerning personal beliefs, religious/spiritual practices, and perceived helpfulness were answered directly by the participants themselves; family members did not provide proxy responses to these subjective items.

### 2.3 Statistical analysis

The sample size was estimated using PASS version 15.0.5 based on the precision of a single proportion. Based on previous research among Chinese patients with schizophrenia, the expected prevalence of religious affiliation was set at approximately 40% (27). Using the exact Clopper–Pearson method, with a two-sided 95% confidence level and a target confidence interval width of 0.12, the required sample size was estimated to be 271 participants (35). Allowing for an anticipated response rate of 90%, the target recruitment sample was approximately 300 participants.

Descriptive statistics were used to summarise the sociodemographic and clinical characteristics of the sample, religious affiliation, religious, spiritual, or folk healing practices, and patient-reported perceived helpfulness of these practices. Continuous variables were summarised using means and standard deviations or medians and interquartile ranges, as appropriate. Categorical variables were summarised using frequencies and percentages.

Classification tree analysis (CTA) was performed to identify factors associated with involvement in religious, spiritual, or folk healing practices. CTA was selected because it is a useful exploratory method for identifying subgroups and interactions among predictors, and it provides an intuitive visual structure for understanding how demographic, clinical, religious/spiritual, and health-belief variables may jointly relate to an outcome (36). This approach is particularly appropriate for culturally oriented clinical research in which relationships between beliefs, practices, and treatment behaviors may be non-linear or dependent on combinations of factors.

Specifically, the Chi-square Automatic Interaction Detection (CHAID) growing method was used to perform the CTA. The significance level for splitting nodes and merging categories was set at P < 0.05. The maximum tree depth was set at four, and the minimum number of cases in parent and child nodes was set at 60 and 30, respectively. The target dependent variable in the first classification tree was the use of any religious, spiritual or folk healing practice. Input variables included sociodemographic characteristics, clinical characteristics, religion-related variables, family supernatural beliefs, and non-conventional health belief scores.

In the classification tree, interactions between factors were interpreted as occurring when a lower-level split depended on the subgroup created by a higher-level split. The classification tree automatically identified variables associated with the target outcome from the root node to terminal nodes, providing an ordered structure of predictor importance and subgroup classification (37). Among participants who reported using religious, spiritual, or folk healing practices, the same CTA procedure was used to identify factors associated with patient-reported perceived helpfulness of these practices.

All analyses were conducted using IBM SPSS Statistics, version 22.0, SPSS Inc., Chicago, IL, USA. A two-sided P value <0.05 was considered statistically significant.

## 3 Results

### 3.1 Demographic and clinical characteristics

Among the 299 patients who completed the survey, 50.5% (151/299) were men, and the mean age was 31.6 years (SD = 10.6). The mean duration of schizophrenia was 6.2 years (SD = 9.2). Overall, 36.5% (109/299) of participants reported a religious affiliation. The most common affiliations were Buddhism (20.1%, 60/299), Christianity (6.0%, 18/299), and folk religion (4.7%, 14/299). Additional demographic characteristics, clinical characteristics, and religion-related beliefs are presented in **Table 1**.

**Table 1:** Characteristics of the sample of patients with schizophrenia (n=299).

Variable	Category	n (%)
Sex	Male	151 (50.5)
	Female	148 (49.5)
Marital status	Married	79 (26.4)
	Never married	169 (56.5)
	Remarried	3 (1.0)
	Co-habitation	7 (2.3)
	Separated or divorced 40	40 (13.4)
	Widowed	1 (0.3)
Occupation	Farmer/fisherman	15 (5.0)
	General worker/service worker	71 (23.7)
	Professional & technical staff/administrative manager	63 (21.1)
	Self-employed/temporary worker	19 (6.4)
	Retired/medically retired	20 (6.7)
	Unemployed/not in employment	101 (33.8)
	Homemaker	10 (3.3)
Religious affiliation	None	190 (63.5)
	Catholicism	7 (2.3)
	Christianity	18 (6.0)
	Buddhism	60 (20.1)
	Taoism	2 (0.7)
	Islam	8 (2.7)
	Folk religion	14 (4.7)
Belief in reincarnation and karma	Yes	120 (40.1)
Having family members with supernatural beliefs	Yes	136 (45.5)
Family history of mental illness	Yes	53 (17.7)
Continuous variables		
Variable	Mean ± SD	Range
Age (years)	31.6±10.6	18-60
Education year	10.9 ± 3.0	4-17
Non-conventional health belief score	12.3±5.8	4-28
Age at onset (years)	24.3 ± 7.6	7-49
Illness duration	6.2 ± 9.2	0-46

### 3.2 Utilization and perceived helpfulness of healing practices

Overall, 57.2% (171/299) of participants reported having ever used at least one religious, spiritual or folk healing practice to cope with or alleviate their illness. Use of religious, spiritual, or folk healing practices was more common among participants reporting a religious affiliation than among those without a religious affiliation (66.1% [72/109] vs. 52.1% [99/190]; χ² = 5.51, P = 0.019). The most commonly reported practice was Zen meditation (44.8%, 134/299), followed by scripture chanting and religious rituals (24.1%, 72/299), religious prayer (14.0%, 42/299), and shamanic dance or ritual practices for exorcism (8.7%, 26/299). As shown in **Table 2**, patterns of specific practice use differed across religious affiliation groups. Zen meditation was most common among participants with Buddhism (65.0%, 39/60), whereas religious prayer was most common among those with Christianity (44.4%, 8/18). Participants without a formal religious affiliation also reported Zen meditation (38.9%, 74/190) and scripture chanting and religious rituals (22.1%, 42/190). Among patients who had used these practices, 69.6% (119/171) perceived them as helpful for their illness. The proportion reporting perceived helpfulness ranged from 57.7% (15/26) to 77.6% (104/134) across the four practices, as shown in **Table 3**.

**Table 2:** Distribution of religious, spiritual, or folk healing practices by religious affiliation.

Religious affiliation	n	Zen meditation	Scripture chanting and religious rituals	Religious prayer	Shamanic dance for exorcism	Any religious healing practice
Buddhism	60	39	26	10	8	46
Christianity	18	7	3	8	2	12
Folk religion	14	7	0	0	0	7
Islam	8	3	0	0	0	3
Catholicism	7	3	0	0	0	3
Taoism	2	1	1	1	0	1
None	190	74	42	23	16	99

**Table 3:** Use and patient-reported helpfulness of religious, spiritual, or folk healing practices among patients with schizophrenia.

Practice	n	%	Perceived helpfulness: Yes	
			n	%
Any religious healing practice	171	57.2	119	69.6
Zen meditation	134	44.8	104	77.6
Scripture chanting and religious rituals	72	24.1	44	61.1
Religious prayer	42	14.0	25	59.5
Shamanic dance for exorcism	26	8.7	15	57.7

### 3.3 Factors associated with the use and helpfulness of healing practices

As shown in **Figure 1**, CTA identified several factors associated with the use of religious, spiritual, or folk healing practices. The first split was based on belief in reincarnation and karma: 76.7% (92/120) of participants who endorsed this belief reported using these practices, compared with 44.1% (79/179) of those who did not (P < 0.001). Among participants who endorsed reincarnation and karma, subsequent splits involved educational level and age; among those who did not, subsequent splits involved non-conventional health beliefs, family members’ supernatural beliefs, and occupation.

**Figure 1:**
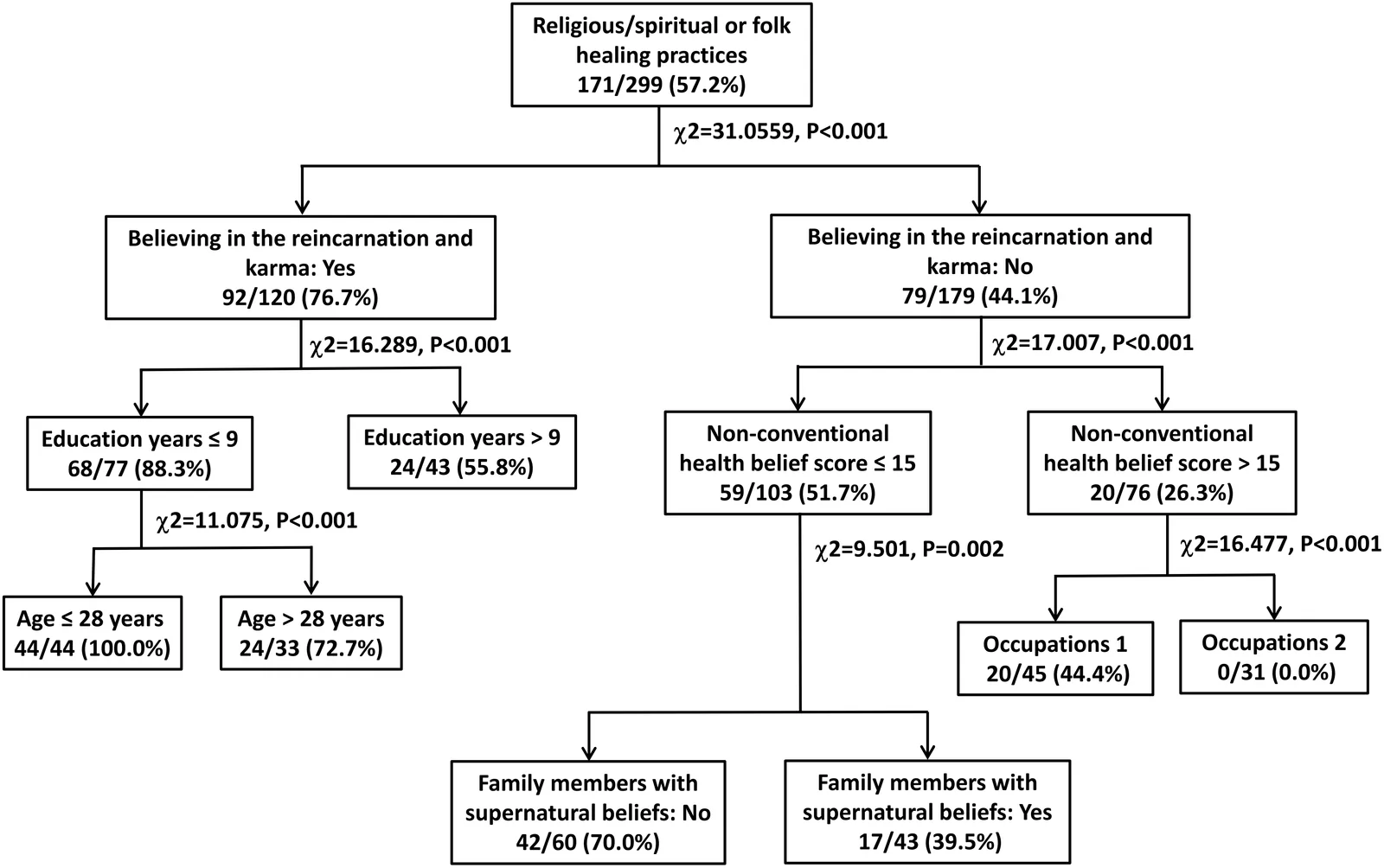
Classification tree analysis of factors associated with religious, spiritual, or folk healing practice use among patients with schizophrenia (N = 299). Each node shows the number and percentage of participants reporting practice use (n/N, %). The χ² statistics and P values indicate the significance of each split.

Among participants who had used religious, spiritual, or folk healing practices, the first split for perceived helpfulness was based on religious affiliation (**Figure 2**). Perceived helpfulness was reported by 92.5% (49/53) of participants affiliated with Buddhism, Daoism, Catholicism, or Islam, compared with 59.3% (70/118) of the remaining participants (P < 0.001). In the latter group, perceived helpfulness was further differentiated by family members’ supernatural beliefs (70.8% [34/48] vs. 51.4% [36/70], P = 0.035).

**Figure 2:**
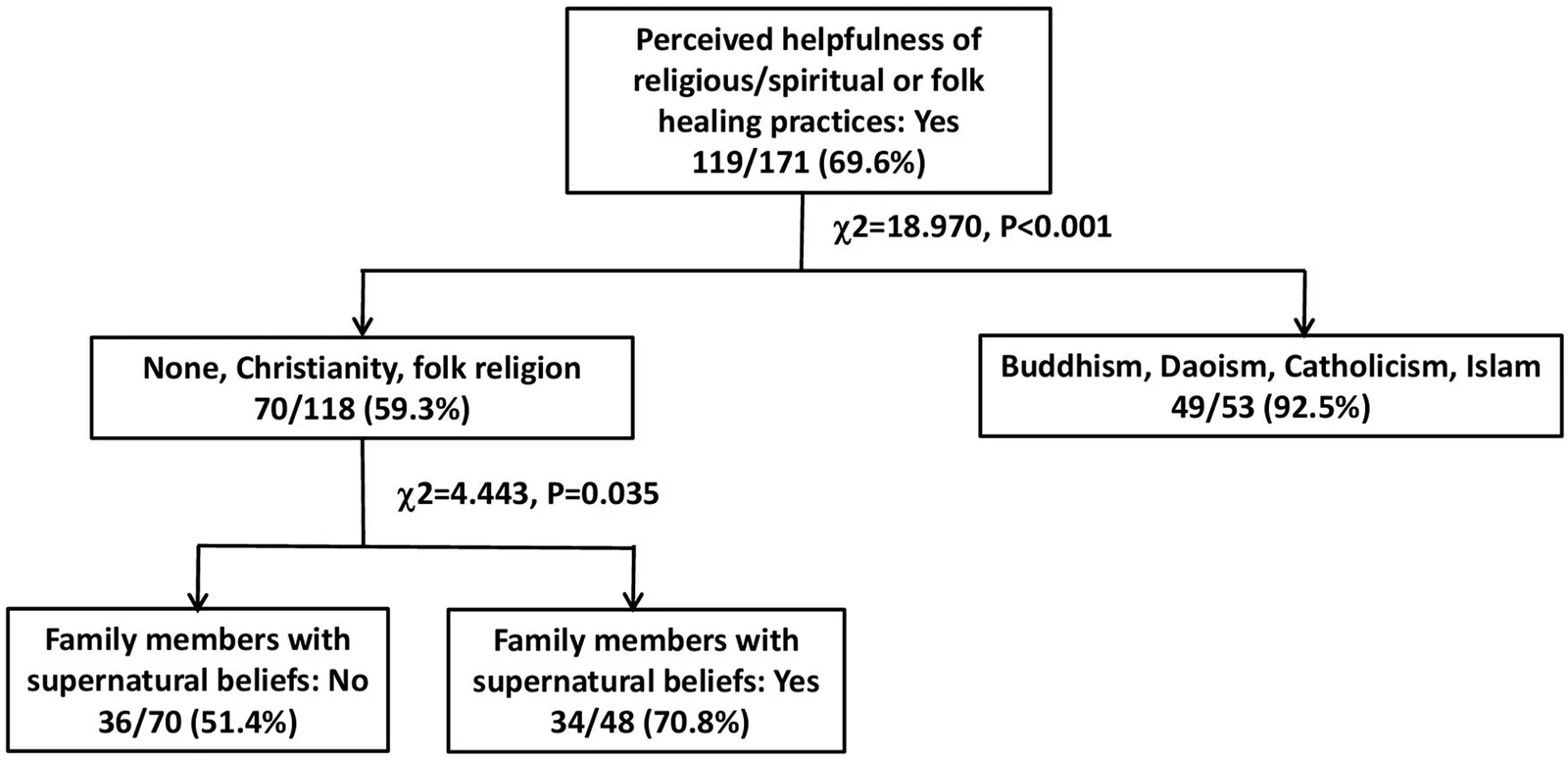
Classification tree analysis of factors associated with perceived helpfulness of religious, spiritual, or folk healing practices among participants who used these practices (n = 171). Each node shows the number and percentage of participants reporting perceived helpfulness (n/N, %). The χ² statistics and P values indicate the significance of each split.

## 4 Discussion

### 4.1 Prevalence and cultural context of religious coping

This study provides insight into the role of religion and spirituality in the illness experiences of Chinese individuals with schizophrenia, particularly in relation to religious affiliation, the use of religious, spiritual, or folk healing practices, and their perceived helpfulness. Within this context, our results indicate that a substantial proportion of participants drew on religious frameworks, particularly those associated with Buddhism, to understand and cope with their illness. This study offers a culturally specific lens on the intersection of religion, spirituality, and schizophrenia in China, a context markedly different from the predominantly Western settings where most research on this topic has been conducted. In addition, one of the methodological merits of this study is the use of CTA, which, unlike traditional logistic regression, provides a more intuitive and hierarchical model for understanding the complex interactions between predictive factors.

The most striking finding of this study is the high prevalence of religious engagement among Chinese patients with schizophrenia. We found that 36.5% of participants reported a religious affiliation, a figure more than double the 14.3% of the general population who claim a formal religious belief, according to China’s 2018 government white paper on religious freedom (38). In addition, 57.2% of patients had engaged in religious, spiritual, or folk healing practices to cope with or alleviate their illness. Although practice use was significantly more common among participants with a religious affiliation than among those without one (66.1% vs. 52.1%), more than half of participants without a formal religious affiliation also reported using these practices. This prevalence of practice use is broadly consistent with two previous regional studies in China, which reported rates ranging from 55.4% to 86.5% (39, 40).

Furthermore, among those who engaged in these practices, 69.6% perceived them as helpful. This level of patient-reported helpfulness should be interpreted as reflecting patients’ subjective illness experience rather than objective clinical efficacy. Our classification tree analyses identified several factors associated with practice use, including belief in reincarnation and karma, non-conventional health beliefs, and family members’ supernatural beliefs. Perceived helpfulness was likewise associated with religious affiliation and family members’ supernatural beliefs. Taken together, these findings suggest that the use and perceived helpfulness of religious, spiritual, or folk healing practices are related to patients’ personal and familial belief contexts.

The unexpectedly high rates of religious belief and practice in our sample may be understood through several intersecting lenses. One possible explanation, proposed in previous literature, is the “religious coping mobilization effect,” whereby religious coping may become more salient during periods of substantial distress (41, 42). Religious and spiritual resources may provide meaning, comfort, and hope for some individuals coping with severe mental illness (43, 44). A second consideration is the Chinese cultural context. Previous literature has described how Buddhist, Daoist, and folk beliefs may provide culturally familiar explanatory frameworks for misfortune and illness (14). Concepts such as karmic retribution, spirit possession, or imbalance of cosmic energies may therefore shape how some individuals interpret experiences associated with psychosis (17, 45). A third contextual consideration is that previous Chinese studies have suggested that mental illness stigma and concerns about “face” may influence patterns of formal and informal help-seeking (46–48). Religious rituals and traditional healing practices may consequently form part of the broader range of culturally familiar resources available to some patients and families (21, 49). However, the present study did not directly assess psychological distress, stigma, motivations for practice use, or pathways to psychiatric care. These explanations should therefore be regarded as possible contextual interpretations derived from previous literature rather than mechanisms demonstrated by the present findings.

### 4.2 Deciphering perceived helpfulness

A notable aspect of our findings is the specific religious landscape of the participants. In the full sample, Buddhism was by far the most prevalent (20.1%), followed by Christianity (6.0%), and folk religions (4.7%). The predominance of Buddhism is unsurprising given its long history and deep integration into Chinese culture. Core Buddhist concepts, particularly karma and reincarnation, provide a powerful explanatory framework for suffering, allowing patients and families to interpret schizophrenia not as a random biological event but as related to past actions, which in turn may offer a pathway for coping through spiritual practice (14). The presence of Christianity and folk religions reflects the syncretic nature of Chinese spirituality. While Christianity is a minority faith, its growth has been documented in China (50). Folk religions, though less frequently claimed as a formal affiliation, are culturally pervasive and often blend seamlessly with Buddhist or Daoist practices, suggesting their influence may be even greater than the numbers indicate (15). This specific blend of beliefs may help shape the types of religious or spiritual practices used, from Zen meditation and scripture chanting rooted in Buddhism to prayer in Christianity and shamanic or ritual practices for exorcism in folk traditions.

The finding that nearly 70% of users perceived religious, spiritual, or folk healing practices as helpful is important but should be interpreted cautiously from a conventional medical perspective, especially for practices such as shamanic or ritual exorcism. This high level of perceived helpfulness should not be equated with clinical efficacy, nor does it indicate improvement in the core psychopathology of schizophrenia (51). Instead, it highlights the crucial distinction between a psychiatrist-assessed clinical outcome, such as symptom reduction measured by the Positive and Negative Syndrome Scale (PANSS), and a patient-reported subjective outcome. The helpfulness reported by our participants likely encompasses a range of psychological, social, cultural, and existential benefits that are not captured by standardised assessments of psychopathology.

Previous studies have suggested that religious and spiritual practices may provide patients with a sense of meaning and purpose, helping some individuals make sense of difficult illness experiences within a spiritual framework (3, 52). This meaning-making is a core component of recovery and has been shown to improve quality of life, even if psychotic symptoms persist (4, 45). Furthermore, religious practices can instill a sense of agency and control, offer hope, and provide comfort, all of which may reduce illness-related distress (43). It is also noteworthy that the most common practice, Zen meditation (44.8%), has a recognised evidence base for alleviating anxiety and depressive symptoms, which are often comorbid with schizophrenia and contribute significantly to a patient’s distress (53, 54). Such psychological benefits may be relevant to patients’ subjective perceptions of helpfulness. Finally, expectancy effects, amplified by deeply held cultural beliefs and strong expectations of healing, may also contribute to the perceived benefits of these ritualistic interventions.

### 4.3 Factors associated with practice use and clinical implications

Our CTA further elucidates patterns associated with the use of religious, spiritual, or folk healing practices by revealing complex interactions between personal beliefs, family context, and sociodemographic factors. The analysis shows that different factors become salient depending on the patient’s core worldview. The first split in the classification tree was belief in reincarnation and karma. Belief in karma may provide a supernatural explanatory framework for the illness, while non-conventional health beliefs, such as beliefs in vital forces and cosmic balance, may offer a compatible framework for understanding and responding to illness (14, 55). However, this belief did not act in isolation. Among those endorsing karmic beliefs, individuals with lower educational attainment (≤9 years) were significantly more likely to engage in religious, spiritual, or folk healing practices. Educational attainment may capture broader differences in mental health literacy, healthcare access, and socioeconomic circumstances that shape how patients respond to illness.

A different pattern emerged among those who did not endorse a karmic worldview. In this group, non-conventional health beliefs emerged as an important correlate of practice use. As shown in **Figure 1**, there is a notable interaction for the subgroup of patients who, despite not believing in karma, hold some non-conventional health beliefs but lack family members’ supernatural beliefs. Within this specific context, occupation becomes a significant factor: individuals employed as general workers or who are unemployed are more likely to report using religious, spiritual, or folk healing practices. These findings suggest that, in the absence of a strong pre-existing supernatural belief system, socioeconomic factors and non-conventional health beliefs may jointly shape the use of religious, spiritual, or folk healing practices.

These factors also be shaped by the family context (56). The presence of family members with supernatural beliefs may serve as a reinforcing social influence, supporting the patient’s worldview and making religious or spiritual help-seeking more acceptable as a course of action (6). This interaction suggests that while personal belief may be the catalyst, it is the sociocultural environment, shaped by education, occupation, and family, which may contribute to the use of religious, spiritual, or folk healing practices.

The factors associated with patient-reported helpfulness, namely having a formal religious affiliation and family members with supernatural beliefs, further illuminate this dynamic. For individuals who already identify with a religion, predominantly Buddhism in our sample, the practices are not foreign interventions but are integrated into an existing framework of meaning, which likely enhances their perceived value. The family’s beliefs, again, may serve as a reinforcing influence, supporting the patient’s experience of the practice as helpful and meaningful. This underscores the important role of the family not only in shaping responses to care but also in contributing to how healing experiences are interpreted.

Our finding that family members’ supernatural beliefs were associated with practice use and perceived helpfulness suggests that the family context may be relevant to how patients engage with and appraise religious or spiritual practices (57). In China’s collectivistic social context, major life decisions, including those related to health, are often made with substantial family involvement (58). Previous studies have suggested that families may play an important role in help-seeking decisions, with their religious beliefs and interpretations of illness potentially influencing the types of care or support pursued (6, 59). Mental illness stigma and concerns about “face” may further shape these decisions by making some families more inclined to seek culturally familiar or less stigmatizing forms of support (47, 60, 61). Accordingly, consultation with local temples or folk healers may form part of the broader help-seeking pathways described in previous Chinese studies (21, 62). Such pathways have been suggested to contribute to delays in accessing psychiatric care in some settings, although stigma, pathways to care, and duration of untreated psychosis were not directly assessed in the present study.

The findings also highlight a potential cultural and religious gap between patients and mental health providers in China (63). The country’s mental health system, largely shaped by biomedical approaches, may not routinely address or engage with the spiritual and folk beliefs held by some patients (29, 64, 65). This may resemble the “religious gap” observed between clinicians and patients in Western settings (43), while taking a distinctive form in China because of differences in religious and cultural traditions (66). When clinicians lack cultural competence and either ignore or pathologize a patient’s deeply held beliefs, it may invalidate their experience, erode trust, and contribute to treatment disengagement (30).

### 4.4 Limitations

This study has several important limitations that should be considered when interpreting the findings. First, the primary outcome, perceived helpfulness, was assessed using a subjective, dichotomous patient-reported measure. Although this measure captures patients’ perspectives, it does not provide an objective assessment of clinical change. The study did not include clinician-rated outcomes using standardised instruments, such as the Positive and Negative Syndrome Scale (PANSS), to assess changes in psychotic symptoms. Therefore, the findings should not be interpreted as evidence that religious, spiritual, or folk healing practices affect the core psychopathology of schizophrenia. Second, we did not collect detailed psychopathological data or standardised measures of symptom severity, nor did we collect detailed treatment information, such as antipsychotic use, medication adherence, or psychotherapy. This limited our ability to examine whether these clinical characteristics were associated with the use or perceived helpfulness of religious, spiritual, or folk healing practices. Third, religious and spiritual involvement was assessed using religious affiliation and several specific belief items rather than validated multidimensional measures. Moreover, the study-specific religious/spiritual practice checklist and non-conventional health belief scale lacked formal psychometric validation. The latter was a preliminary four-item measure supported only by internal consistency in the present sample, and its factor structure and validity require further evaluation and refinement in future studies. Fourth, the cross-sectional design precludes causal inferences regarding the relationships between religious beliefs, practice use, and perceived helpfulness. Fifth, the CTA was exploratory and was intended to identify potential subgroup patterns and interactions rather than to develop a predictive model. The stability and generalizability of the identified tree structures therefore require confirmation in independent samples. Finally, the recruitment of clinically stable outpatients aged 18–60 years from a single tertiary psychiatric hospital may limit the generalizability of the findings to older adults, inpatients, patients with acute psychosis, treatment-naïve patients, and populations in rural areas or regions with different religious traditions.

## 5 Conclusion

Religious and spiritual complementary practices were commonly reported among Chinese outpatients with schizophrenia and were perceived as helpful by a substantial proportion of those who used them. Classification tree analysis further identified distinct patterns associated with practice use and perceived helpfulness, highlighting the roles of personal beliefs, religious affiliation, family members’ supernatural beliefs, and selected sociodemographic characteristics. These findings underscore the importance of considering patients’ religious and spiritual beliefs and practices, together with their family and sociocultural contexts, in routine psychiatric assessment and communication. Greater awareness of these factors may contribute to more culturally responsive and spiritually informed psychiatric care in China.

## 6 Statements

### 6.1 Data availability statement

The datasets used and/or analysed during the current study are available from the corresponding author on reasonable request.

### 6.2 Ethics statement

The study was conducted in accordance with the Declaration of Helsinki. The study protocol and informed consent procedures were approved by the Medical Ethics Committee of Wuhan Mental Health Center (Approval No. KY2024.0227.01). Written informed consent was obtained from all participants. Participants’ capacity to provide informed consent was assessed by their treating psychiatrists. For 12 participants (4.0%), additional written consent from a legal guardian was obtained in accordance with institutional ethical requirements. Guardian consent served as an additional ethical safeguard and did not involve proxy completion of the questionnaire.

## Data Availability

The raw data supporting the conclusions of this article will be made available by the authors, without undue reservation.
